# Controlled dimerization of artificial membrane receptors for transmembrane signal transduction†

**DOI:** 10.1039/d1sc00718a

**Published:** 2021-05-05

**Authors:** Hui Chen, Li Zhou, Chunying Li, Xiaoxiao He, Jin Huang, Xiaohai Yang, Hui Shi, Kemin Wang, Jianbo Liu

**Affiliations:** State Key Laboratory of Chemo/Biosensing and Chemometrics, College of Biology, Key Laboratory for Bio-Nanotechnology and Molecular Engineering of Hunan Province, Hunan University Changsha 410082 P. R. China liujianbo@hnu.edu.cn huishi_2009@hnu.edu.cn

## Abstract

In biology, membrane-spanning proteins are responsible for the transmission of chemical signals across membranes, including the signal recognition-mediated conformational change of transmembrane receptors at the cell surface, and a trigger of an intracellular phosphorylation cascade. The ability to reproduce such biological processes in artificial systems has potential applications in smart sensing, drug delivery, and synthetic biology. Here, an artificial transmembrane receptors signaling system was designed and constructed based on modular DNA scaffolds. The artificial transmembrane receptors in this system are composed of three functional modules: signal recognition, lipophilic transmembrane linker, and signal output modules. Adenosine triphosphate (ATP) served as an external signal input to trigger the dimerization of two artificial receptors on membranes through a proximity effect. This effect induced the formation of a G-quadruplex, which served as a peroxidase-like enzyme to facilitate a signal output measured by either fluorescence or absorbance in the lipid bilayer vesicles. The broader utility of this modular method was further demonstrated using a lysozyme-binding aptamer instead of an ATP-binding aptamer. Therefore, this work provides a modular and generalizable method for the design of artificial transmembrane receptors. The flexibility of this synthetic methodology will allow researchers to incorporate different functional modules while retaining the same molecular framework for signal transduction.

## Abstract 2

An artificial transmbrane signal transducer was developed through the chemical input-mediated dimerization of artificial DNA transmembrane receptors and the subsequent activation of a cascade of events inside the vesicles.

## Graphical abstract

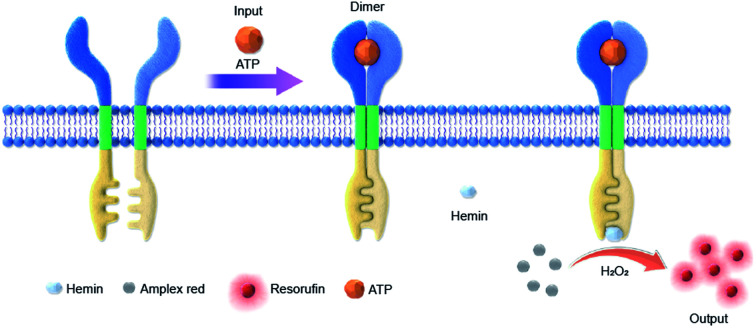

## Introduction

Transmembrane signal transduction is an important feature of biological processes that allows cells to respond to changes in their external environment and communicate among cells.^1,2^ Signal transduction is initiated typically by a hormone or neurotransmitter as first messengers that interact with transmembrane protein receptors on the cell surface, which cause a conformational change in membrane-spanning transmitter units and ultimately trigger an enzymatic reaction cascade and a release of second messenger molecules into the cytosol. In natural systems, two prominent mechanisms of transmembrane signal transduction have evolved: a global conformational change as exemplified by G protein-coupled receptors (GPCRs)^3^ and dimerization of the membrane-spanning receptors as observed in receptor tyrosine kinases (RTKs).^4,5^ In recent years, significant efforts have been made to create various artificial transmembrane systems inspired by their biological counterparts, and such mimics have potential applications in drug delivery, smart sensing, and synthetic biology.^6–8^ There are several reports on artificial signal transduction systems constructed from organic polymers or host–guest supramolecular chemistry.^9–11^ For example, Clayden *et al.* reported an artificial GPCR signaling system in which the binding of specific carboxylate ligands to a Cu(ii) cofactor at the binding site perturbed the foldamer's global conformation.^12^ Schrader *et al.* published an artificial RTK signaling system in which the transmembrane units were constructed from fluorescence resonance energy transfer (FRET) pairs and a bisphosphonate dianion receptor for dimerization.^13–15^ Hunter *et al.* described an alternative translocation mechanism for transmembrane signaling in which a pH-modulated polar switch was coupled to the activation of a catalyst inside a vesicle for fluorescence signal transduction and amplification.^16–19^

Recently, DNA nanostructures have emerged as new molecular scaffolds for mimicking cellular components and processes. The DNA structure might provide a unique tool for the construction of transmembrane mimics because of convenient chemical synthesis, easy programmability, sequence-based design, and facile integration.^20–22^ Most importantly, the geometry and chemical functionality of this novel class of biomimetic systems can be rationally designed utilizing DNA nanotechnology approaches, which enable the complete *de novo* design of biomimetic systems with atomically defined nanostructures.^23,24^ A few artificial transmembrane DNA channels and DNA pores have been reported in which the signal communications mostly relied on mimicking the direct physical transport of signal molecules across the membrane.^25–29^ However, the mimicking of transmembrane signal transduction without physical mass transfer is considerably more challenging. We are currently unaware of any studies that have examined the design and construction of DNA nanostructures from the perspective of artificial transmembrane signal transduction.^30,31^

**Fig. 1:**
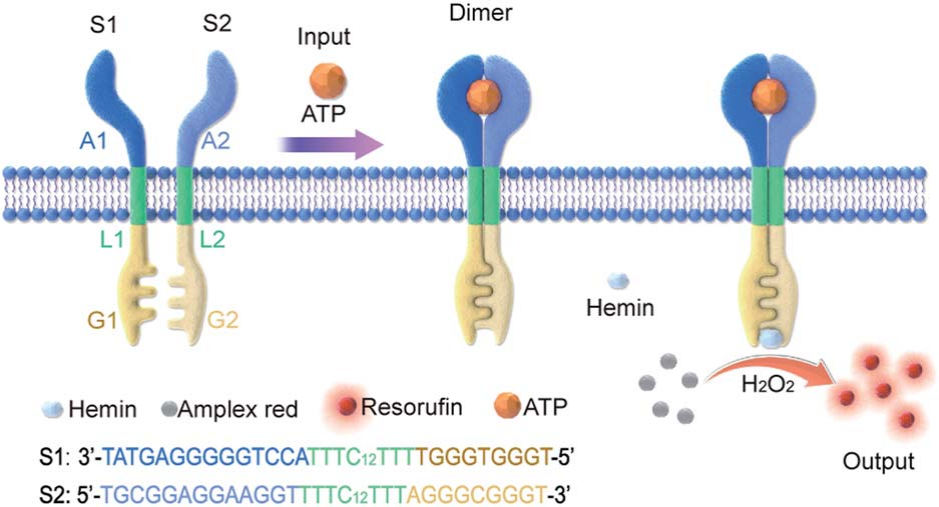
Controlled dimerization of artificial membrane receptors for transmembrane signal transduction. The S1 monomer was composed of three DNA modules: A1, L1, and G1; the S2 monomer was composed of three DNA modules: A2, L2, and G2. While A1 and A2 are the split sequences of an ATP-binding aptamer, G1 and G2 are the split sequences of a G-quadruplex. ATP triggers the oligo-dimerization of artificial DNA receptors on the membrane, which are coupled to the G-quadruplex-mediated peroxidation of Amplex Red to resorufin and fluorescence signal output on the inside of the membrane.

In this work, an artificial transmembrane signal transducer was developed through the chemical input-mediated dimerization of artificial DNA transmembrane receptors and the subsequent activation of a cascade of events inside the vesicles. Our approach involved mimics of transmembrane receptors with two transmembrane units, which carry recognition sites on the external terminus and signaling sites on the internal terminus. As shown in Fig. 1, the artificial transmembrane units had a dimeric structure, and each artificial receptor monomer was designed using three functional modules (Fig. S1†): split adenosine triphosphate (ATP) aptamers as signal recognition modules for the specific binding of input molecules; linker sequences with a 12-carbon (C12) spacer as a lipophilic transmembrane module; and a split G-quadruplex (G4) as a signal output module capable of performing peroxidase-like catalytic reactions. We reasoned that the addition of ATP as a signal input would trigger the dimerization of two artificial receptors on membranes, resulting in the proximity of two monomer sequences and the formation of a G-quadruplex (G4). Subsequently, a G4–hemin complex would serve as a peroxidase-like enzyme to facilitate a signal output, such as a G4–hemin-mediated Amplex Red fluorescent reaction or a 2,2′-azinobis-(3-ethylbenzothiazoline-6-sulphonic acid) (ABTS) colorimetric reaction in the vesicle lumen. G-quadruplex could bind to hemin to form the hemin/G-quadruplex which acts as horseradish peroxidase-mimicking DNAzyme to catalyze the reduction of H_2_O_2_.^32^ The idea is that the intermolecular reaction would be slow, but following dimerization of the external recognition groups, the internal signaling groups are brought into close proximity, which would give rise to fast intramolecular reaction. Here, for the first time, we present a DNA-based model that imitates the transmembrane signal transduction process comprising extracellular receptor recognition, dimerization, and an intracellular catalytic reaction. This work provides a universal and modular approach to the construction of transmembrane receptors, wherein three modules can be replaced by other functional motifs to extend their applications. We demonstrate this flexibility by the substitution of the ATP-binding split aptamer with a split lysozyme-binding aptamer. A distinct protein-responsive transmembrane model of signal transduction was thereby successfully developed.

## Results and discussion

### ATP mediated dimerization of split sequences in homogenous solution

**Fig. 2:**
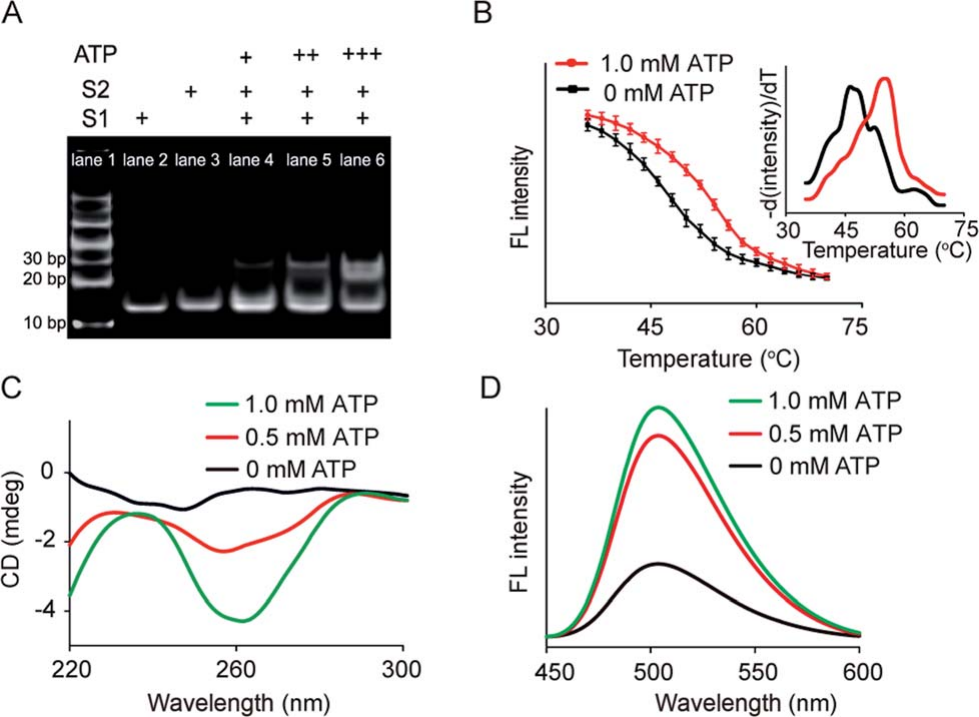
ATP-mediated dimerization of split sequences in homogeneous solution. (A) Page: lane 1, DNA marker; lane 2, S1; lane 3, S2; lane 4, S1 + S2 + ATP (+); lane 5, S1 + S2 + ATP (++); lane 6, S1 + S2 + ATP (+++). S1 and S2 = 1.0 μM. ATP (+) = 0.1 mM, ATP (++) = 0.5 mM, ATP (+++) = 1.0 mM. (B) Melting curves of the split sequence mixture (S1 and S2) in the absence or presence of ATP (1.0 mM). Fluorescence intensity of the split sequence mixture with SYBR Green I as a function of temperature. Inset: Negative first derivative of the melting curves. (C) CD spectra of the split sequence mixture in the presence of ATP. (D) G-quadruplex-induced fluorescence enhancement of ThT resulting from the ATP-mediated dimerization of split sequences.

Artificial membrane receptors composed of two split DNA sequences S1 and S2 (Fig. S1†), which comprised split ATP-binding aptamers, were designed for dimer formation in response to the ATP signal input. Dimerization in homogeneous buffer solution was characterized using polyacrylamide gel electrophoresis (PAGE), which revealed that a new band representing dimers emerged after the addition of ATP to the mixture of split sequences S1 and S2 (Fig. 2A). In a control experiment in which the S2 sequence was replaced by a random sequence (Sr), no obvious dimerization was observed (Fig. S3A†). The melting curves of the mixed sequences were acquired by staining with SYBR Green I and analyzed by negative first derivative fitting (Fig. 2B). The addition of ATP facilitated an increase in the melting temperature (*T*_m_) of the DNA complex from 46.2 °C to 55.0 °C, indicating DNA dimer formation (Fig. 2B). Dimer dimerization was accompanied by the formation of G-quadruplex structures, which was determined by measuring the circular dichroism (CD) spectra. As shown in Fig. 2C, the gradual addition of ATP (0–1.0 mM) to the split sequence mixture (S1 and S2, 1.0 μM) induced the appearance of a new negative peak around 260 nm, corresponding to a characteristic peak of the antiparallel G-quadruplex structure. The formation of G-quadruplex structures was further confirmed by thioflavin T (ThT) fluorescence in which the binding of ThT to G-quadruplex caused the fluorescence enhancement of ThT. The fluorescent emission of ThT at 504 nm increased by 2.2-fold after the addition of ATP (1.0 mM), which was contributed to the formation of the G-quadruplex (Fig. 2D). However, there was no obvious change in CD spectra or ThT fluorescence in the control system in which the split S2 sequence was replaced by a random sequence (Sr) (Fig. S3B and C†). Thus, our results indicated that the addition of ATP led to dimerization, resulting in a G-quadruplex antiparallel structure.

### Transmembrane signal transduction mediated FRET

**Fig. 3:**
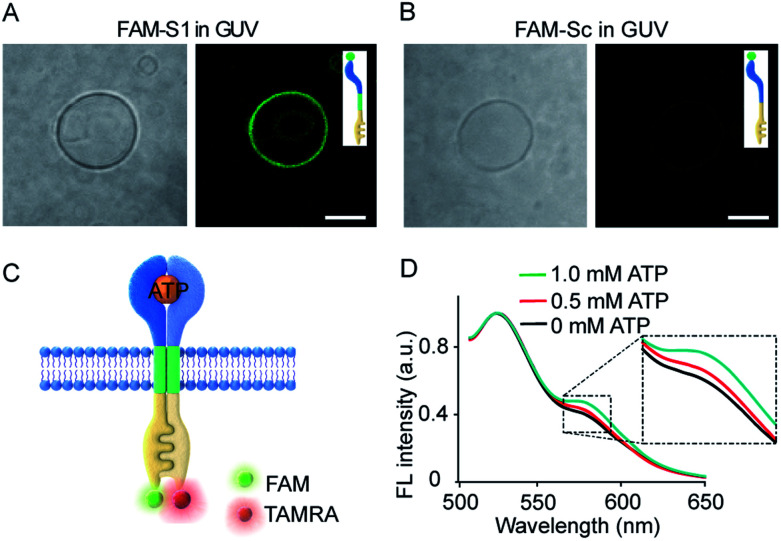
(A and B) Fluorescence images of FAM-S1 (A) and FAM-Sc (B) spanning the membrane of GUVs. Scale bar: 5 μm. (C) Schematic illustration of ATP-triggered dimer formation and transmembrane FRET. S1 and S2 were labelled separately with FAM and TAMRA, respectively. (D) Fluorescence spectra of the FRET pair after the addition of ATP (0–1.0 mM). FAM-S1 : TAMRA-S2 = 1 : 5 molar ratio.

Transmembrane signal transduction mediated FRET. The first requirement for the transmembrane signal transduction was to identify the monomer sequence anchored within the lipid membrane. In our system, a C12 spacer was designed to link the split aptamer modules and split G-quadruplex modules and served as a hydrophobic domain to facilitate the penetration of the artificial receptor monomer into the lipid membrane. Therefore, we used fluorescent imaging to characterize the immobilization of 5-carboxyfluorescein (FAM) labelled S1 (FAM-S1) on giant unilamellar vesicles (GUVs) using FAM-Sc without C12 spacer modification as a control. Low fluorescence signals were detected in phospholipid vesicles containing FAM-Sc (Fig. 3B), whereas mixtures of FAM-S1 and GUV exhibited a significant 5.1-fold increase in fluorescence around the walls of the GUVs (Fig. 3A). These comparisons demonstrated that the hydrophobic modification of monomer sequences facilitated their insertion into lipid bilayers.

Dimerization experiments in membrane were conducted in LUVs (Fig. S4†). S1- and S2-containing vesicles were assembled by hydrating a dried mixture of lipids, S1, and S2 in Tris–HCl buffer (100 mM, pH = 7.0). The suspension was extruded and suspended in buffer solution to result in final bulk concentrations of 2.5 mM lipids and 4 mM transducer monomers. The membrane fluidity of the vesicles was validated using lipophilic pyrene probes based on their spatially dependent excimer spectra (Fig. S5A†). Pyrene-rich LUVs were diluted in a pyrene-free LUV suspension. Pyrene monomer fluorescence at 375 nm increased gradually, accompanied by a decrease in pyrene excimer fluorescence at 475 nm (Fig. S5B†). The spectra fluctuation indicated vesicle fusion and molecule diffusion associated with membrane fluidity.

Given the insertion of the artificial receptor into the membrane and fluidity of the lipid membrane, the addition of ATP to S1- and S2-containing vesicles was expected to trigger the dimerization of split sequences on the membrane. ATP-triggered dimerization would bring the artificial receptor monomers into close proximity. To confirm the dimerization reaction on membranes, a FRET experiment was conducted in the vesicles in which S1 and S2 were labelled with FAM and 5-carboxytetramethylrhodamine (TAMRA), respectively (Fig. 3C). As shown in Fig. 3D, the normalized fluorescence spectra showed that the gradual addition of ATP led to a gradual increase in fluorescence at 585 nm, and the addition of 1.0 mM ATP resulted in an increase in the FRET efficiency (the ratio of *F*_a_/*F*_d_) from 0.4 to 0.5. The occurrence of FRET between donors and acceptors is evidence of their close proximity and is indicative of dimer formation. In a control experiment, no obvious FRET was observed when FAM-S1 was replaced by random sequences containing FAM-Sc (Fig. S6†). Overall, the FRET spectra of vesicles suggested that the addition of ATP facilitated the proximity of receptor monomers and dimer formation on the vesicle membranes.

### Transmembrane transduction mediated signal output

**Fig. 4:**
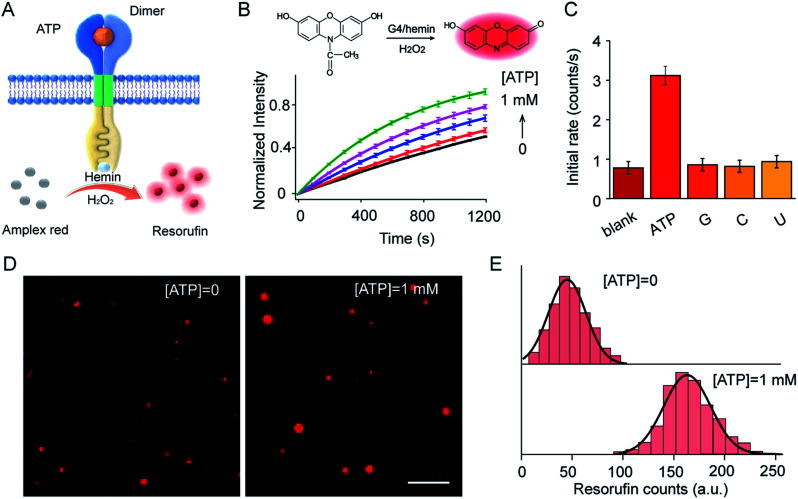
(A) Schematic illustration of ATP-triggered dimer formation and G4–hemin-mediated Amplex Red oxidation. (B) Fluorescence kinetic curves of Amplex Red oxidized by G4–hemin after the addition of ATP (0–1.0 mM). Inset: Schema of G4–hemin-mediated Amplex Red oxidation by H_2_O_2_. S1 : S2 = 1 : 1 molar ratio. H_2_O_2_, 50 μM; hemin, 4.0 μM; Amplex Red, 20 μM. (C) Guanosine (G), cytidine (C), or uridine (U) were used as input instead of ATP to determine selectivity. (D) TIRFM imaging of S1- and S2-containing LUVs before ([ATP = 0]) and after ([ATP] = 1 mM) the addition of ATP. Ex, 532 nm, Em, 573–613 nm. Scale bar: 5 μm. (E) Frequency analysis of fluorescent photon counts from vesicle imaging in (D). *n* = 100–120. *t*-test, *P* = 0.0001 for the sample with ATP compared to that without ATP.

ATP-mediated dimerization was coupled to G4 formation in the interior of the vesicles, which served as a peroxidase-like enzyme for signal production. For example, Amplex Red can be oxidized by H_2_O_2_ to produce highly fluorescent resorufin in the presence of the G4–hemin complex (Fig. 4A). Amplex Red and hemin were encapsulated simultaneously in vesicles during LUV preparation, and the fluorescence kinetics were monitored on the addition of ATP in the presence of H_2_O_2_. As shown in (Fig. 4B), ATP input was associated with the fluorescence enhancement of the vesicles. The introduction of ATP initiated Amplex Red peroxidase oxidation, and the fluorescent intensity at 585 nm increased gradually. Specifically, the initial enzymatic reaction rate increased 4.1-fold on the addition of 1.0 mM ATP compared to that in the absence of ATP (Fig. 4B). However, no obvious fluorescence enhancement was observed after the addition of guanosine, cytidine, or uridine instead of ATP (Fig. 4C). The fluorescence signal output was further visualized using total internal reflection fluorescence microscope (TIRFM) imaging. To recognize the fluorescent signals of single vesicles, imaging from two different fluorescent channels were subjected to threshold analysis. Single LUVs were observed separately and distributed individually (Fig. 4D). The brightness of single vesicles increased robustly after the addition of ATP (Fig. 4D). The fluorescence enhancement was contributed to the G4 formation and catalysis of Amplex Red oxidation in the interior of the vesicles. To quantitate the intensity distribution, vesicle images before and after the addition of ATP were subjected to statistical analysis of frequency to determine the average photon intensity of the fluorescent spots. The introduction of ATP facilitated an increase in fluorescence from 45.3 ± 20.1 to 162.5 ± 25.9 counts (*t*-test, *P* = 0.0001), representing a 3.6-fold fluorescence enhancement (Fig. 4E).

G4–hemin complex-mediated ABTS oxidation was also carried out when ABTS and hemin were encapsulated simultaneously in LUVs. Ultraviolet-visible (UV-vis) adsorption kinetic curves demonstrated that the introduction of ATP initiated ABTS peroxidase oxidation, and adsorption at 420 nm increased gradually (Fig. S7A†). Specifically, the initial enzymatic reaction rate increased 3.7-fold after the addition of 1.0 mM ATP (Fig. S7B†). Overall, dimer-mediated fluorescence and colorimetric signal outputs in the vesicles were demonstrated in this artificial transmembrane system in response to the external ATP input. It is worth pointing out that a high signal background was observed in this system, even in the absence of ATP. We attributed this to the hemin background and random distribution of receptor orientation. Hemin will also facilitate the dimerization of the monomers to produce a fluorescent background. Incorporation of the transducer monomers during vesicle preparation results in the random distribution of monomers in the inner and outer leaflets of the membrane. However, only the transmembrane units in the outer leaflet are in the correct orientation to interact with the external signal input and to catalyze reactions inside the vesicle. Fluorescence vesicle experiments were used to quantify the possible topologies, based on the fluorescence enhancement of ThT upon binding with G-quadruplex. The transmembrane units in the correct orientation accounted for around 33% (Fig. S8†). The remaining other of the transmembrane sequences that were in an inverted orientation were not sensitive to changes in the external ATP input.

### Lysozyme mediated transmembrane signal transduction

**Fig. 5:**
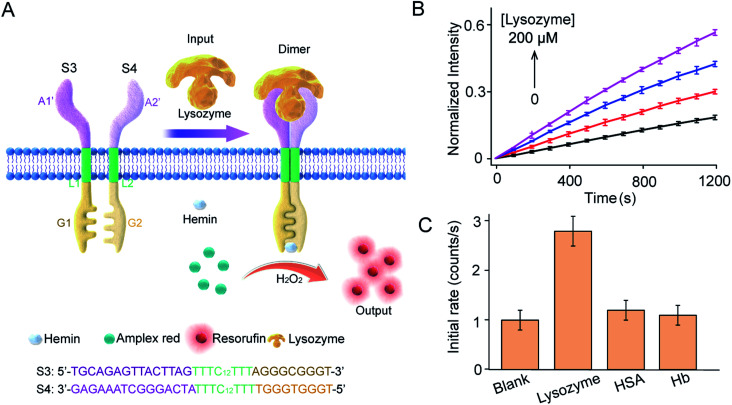
Lysozyme-mediated modular transmembrane signal transduction. (A) Design of membrane receptors and schematic diagram of signal conduction in which lysozyme was used to trigger the dimerization of artificial DNA receptors on membranes. Receptor engagement was coupled to G-quadruplex-mediated peroxidase activity and signal output on the inside of the membrane. S3 and S4 monomers were composed of three DNA modules. The signal recognition modules were split sequences of a lysozyme-binding aptamer. (B) Fluorescence kinetic curves of Amplex Red oxidized by G4–hemin after the addition of lysozyme (0–200 μM). S3 : S4 = 1 : 1 molar ratio. H_2_O_2_, 50 μM; hemin, 4.0 μM; Amplex Red, 20 μM. (C) HSA and Hb were used as signal input instead of lysozyme to determine selectivity.

This work provides a modular approach to the design and construction of transmembrane receptors in which three modules can be replaced by other functional motifs. To confirm this, a signal recognition module was designed using spilt lysozyme-binding aptamers, which can respond to lysozyme protein as an input (Fig. 5A). In homogeneous buffer solution, lysozyme mediated dimerization and G4 formation as confirmed by CD spectra, melting curves, and fluorescence enhancement (Fig. S9†). Lysozyme-mediated transmembrane signal transduction was conducted using S3 and S4 immobilized in substrate-encapsulating vesicles. As demonstrated by fluorescence kinetics curves (Fig. 5B), the introduction of lysozyme initiated Amplex Red peroxidase oxidation, and the fluorescent intensity at 585 nm increased gradually. Specifically, after the addition of 200 μM lysozyme, the initial enzymatic reaction rate increased 2.6-fold compared to that in the absence of lysozyme. However, no obvious fluorescence enhancement was observed after the addition of human serum albumin (HSA) or hemoglobin (Hb) instead of lysozyme (Fig. 5C). Compared to the ATP synthetic system, a lower concentration of lysozyme was required to trigger signal production in the vesicles, which was attributed to the smaller dissociation constant of the lysozyme-binding unsplit aptamer (*K*_d_ = 31 nM)^33^ than that of the ATP binding unsplit aptamer (*K*_d_ = 6 μM).^34^ Therefore, the versatility of this modular method was well demonstrated.

## Conclusions

Biological signaling events in which information is transmitted from the exterior to the interior of cells are critical for life. Developing synthetic signaling systems that allow the controlled and detailed investigation of such processes may provide valuable information about how nature regulates these events. Here, we demonstrate the first example of artificial transmembrane signal transduction in a DNA-based synthetic system. Using DNA as a material to construct artificial receptors may be an apt method for the study of intracellular and extracellular communication. The modular design of this system could be expanded easily to any target of interest by simply changing the recognition motifs. The dimerization of two receptors for controllable signal transduction shows excellent versatility for potential applications. For example, the flexibility of the recognition modules of DNA receptors, including those recognizing aptamers and DNAzymes, allows for a wide range of small molecules or light stimuli as specific inputs. The signal output modules in the interior could also be replaced by split FRET probes or split enzymes to extend their applications.

This artificial cascade mimics the membrane-spanning proteins signaling mechanism of coupling with separate chemistry on the inside and outside of membranes. In contrast to transport mechanisms, the message and signal can be chemically unrelated using this mechanism. This synthetic approach may be applied to designing a new class of chemical tools that can control the activities of native cells without physical transmembrane transport. Therefore, this work provides the impetus to widen the scope of this methodology to create vesicles responsive to diverse chemical signals and ultimately provide the basis to develop bioinspired nanotechnologies capable of interfacing and exchanging information with biological systems.

## Experimental section

### Materials and reagents

DNA sequences were synthesized and purified using high-performance liquid chromatography by Sangon Biotech and used after dissolving in Tris–HCl buffer. Adenosine triphosphate (ATP), lysozyme, hemin, Amplex Red, 1,2-dipalmitoyl-*sn*-glycero-3-phosphocholine (DPPC) and egg yolk phosphatidyl choline (EYPC) were purchased from Sigma-Aldrich. SYBR Green I, ABTS, thioflavin T (ThT), hemoglobin (Hb), and human serum albumin (HSA) were purchased from Sangon Biotech. H_2_O_2_ was purchased from Sinopharm. Ultrapure water was obtained using a Millipore water purification system (18.2 MΩ cm^−1^). All reagents were obtained from commercial suppliers and used directly for the following experiments without further purification, unless otherwise noted.

Ultraviolet-visible (UV-vis) measurements were performed using a Shimadzu UV-2600 spectrophotometer (Shimadzu).

Fluorescence measurements were performed using an F-7000 fluorimeter (Hitachi) equipped with a xenon lamp at room temperature. Circular dichroism (CD) was performed using a Biologic MOS-500 (Bio-Logic). Gel images were obtained using a gel imaging system (Azure Biosystem). The vesicles were extruded using a MiniExtruder (Avanti Polar Lipids) to homogenize vesicles. Confocal fluorescence images were acquired on a confocal laser scanning microscope (Nikon A1). Fluorescence images of single vesicles were acquired on a total internal reflection fluorescence microscope (TIRFM) (Nikon TE2000). Dynamic light-scattering measurements were performed using a Zetasizer 3000Hs (Malvern).

### ATP-mediated dimerization of split sequences in homogeneous solution

DNA was heated to 95 °C for 5 min and cooled on ice for 1 h before use. The artificial receptor sequences S1 and S2 (Fig. 1) were mixed at an appropriate molar ratio (S1 : S2 = 1 : 1, 1.0 μM) in Tris–HCl buffer (100 mM, pH = 7.4), followed by the addition of different concentrations of ATP (0–1.0 mM) and incubation for 1 h at 37 °C. ATP-mediated dimerization was analyzed using 15% native polyacrylamide gel electrophoresis (PAGE). Tris/borate/EDTA (TBE) buffer (1× TBE) was used as an electrophoresis running buffer, and the gel was run at 80 V (constant voltage) for 2 h. The buffer temperature was controlled to maintain the samples at 4 °C throughout electrophoresis. After electrophoresis, the gel was stained with SYBR Gold and imaged using a gel imaging system. ATP-mediated dimerization was characterized using CD spectrometry from 220 to 320 nm. The step size was set to 1 nm after correcting for the solvent background. Dimerization (S1, S2 = 1.0 μM) was also characterized using a fluorescence spectrometer. After the addition of ATP to the DNA mixture, ThT solution (20 μM) was added and incubated for 1 h. Fluorescence spectra were measured using excitation at 425 nm and emission from 450 to 600 nm. The thermal stability of the resultant dimer was analyzed using an F-7000 instrument by mixing the DNA dimer complex (S1, S2 = 1.0 μM; ATP = 1.0 mM) with SYBR Green I (1 μM). The fluorescence data for melting curves were acquired at increments of 1 °C during transition from 35 °C to 70 °C (15 s at each temperature). The fluorescence data were then converted to melting peaks and plotted as the negative derivative of fluorescence intensity as a function of temperature.

### Preparation of unilamellar vesicles and membrane insertion investigation

Giant unilamellar vesicles (GUVs) were prepared using a dry-film hydration method. EYPC (4.5 mg) and DPPC (0.5 mg) were mixed in 3 mL of a chloroform/methanol (1 : 2, v/v) solution. The lipids were deposited onto the bottom of the glass surface of a 25 mL round-bottom flask after rotating evaporation for 24 h. Hydration of the lipid film was initiated by the addition of an aqueous solution, which was poured gently down the side of the flask at 40 °C. The lipid film was allowed to swell for 2 h or more, which led to the formation of GUVs. The GUVs were suspended in 100 mM of Tris–HCl buffer (pH 7.0) and imaged using a confocal microscope with a 100× oil immersion objective in bright-field and fluorescence modes. While imaging, FAM-S1 was added at a concentration of 5 nM, and FAM-Sc served as a control. FAM was excited at 514 nm and emission above 530 nm was collected. Images were processed using ImageJ.

Large unilamellar vesicles (LUVs) were prepared using a dry-film hydration method. DPPC (10 mg) in 1 mL of a chloroform solution was added to an oven-dried round-bottom flask (10 mL). The solvent was removed under vacuum using a rotary evaporator for 20 min, after which the thin film was dried under ultrahigh vacuum for 3 h. Hydration of the lipid film was initiated by the addition of an aqueous solution, and the solution was then sonicated for 20 min at room temperature. LUVs were stored in the fridge and used within 1 week. Samples were pipetted into a 1.5 mL microcentrifuge tube and then extruded through a 200 nm polycarbonate membrane (Whatman) to homogenize the vesicles.

### Transmembrane signal transduction-mediated FRET in LUVs

S1 and S2 were labelled with FAM and TAMRA, respectively, and the FRET assay was used to confirm transmembrane signal transduction. To facilitate the insertion of DNA sequences into the lipid membrane, FRET sequences were synchronously mixed in the lipid solution to prepare the vesicles. Therefore, FAM-S1- and TAMRA-S2-containing LUVs were prepared by synchronously mixing FAM-S1 and TAMRA-S2 (FAM-S1 : TAMRA-S2 = 1 : 5 molar ratio) in the lipid solution. Different amounts of ATP (0–1.0 mM) were added to the vesicle suspensions as an external signal input and their fluorescent spectra were determined with excitation at 488 nm and emission at 500–650 nm.

### Transmembrane signal transduction-mediated peroxidase reaction

To facilitate the insertion of DNA sequences into the lipid membrane, S1 and S2 sequences (4 μM) were synchronously mixed in the lipid solution to prepare the vesicles. Meanwhile, a Tris–HCl buffer solution (100 mM, pH = 7.0, 20 μM Amplex Red and 4 μM hemin) was used for the hydration of the lipid film so that the dyes were encapsulated in the vesicles. ATP (0–1.0 mM) was added as an external signal input and incubated for 1 h. To investigate recognition selectivity, guanosine (G), cytidine (C), or uridine (U) were added as input instead of ATP. The above vesicle samples were diluted 10 000-fold for TIRFM imaging with 488 nm laser excitation. Amplex Red fluorescence data were collected using a Cy3 filter. Meanwhile, the fluorescence signal at 585 nm was monitored after the addition of H_2_O_2_ (50 μM). Alternatively, UV-vis adsorption at 420 nm was monitored when 20 μM Amplex Red was replaced by 4 mM ABTS.

### Lysozyme-mediated transmembrane signal transduction

To test the versatility of this system, we replaced the split ATP-binding aptamer with the split lysozyme-binding aptamer. CD spectra, melting curves, and fluorescence enhancement experiments were conducted in homogeneous solution as with the ATP system. However, S1 and S2 were replaced by S3 and S4 (Fig. S1†). Then, the fluorescence kinetic curve was used to characterize signal transmission. S3 and S4 sequences (4 μM) were synchronously mixed in the lipid solution to prepare the vesicles, and the resulting LUVs were suspended in 100 mM Tris–HCl buffer solution (pH = 7.0, 20 μM Amplex Red and 4 μM hemin). Lysozyme (0–200 μM) was added as an external signal input, and the fluorescence signal at 585 nm was monitored after the addition of H_2_O_2_ (50 μM).

## Author contributions

J. L. and H. S. conceived and supervised the project. H. C., L. Z., and K. W. designed the experiments. L. Z. and H. C. performed the experiments and analyzed the data. C. L. performed confocal laser scanning microscope imaging. X. H., J. H., and X. Y. provided experimental advice. H. C. and L. Z. wrote the paper.

## Conflicts of interest

There are no conflicts to declare.

## Supplementary Material

SC-012-D1SC00718A-s001

## References

[cit1] Krauss G., Biochemistry of Signal Transduction and Regulation, 3rd edn, Wiley-VCH, 2006

[cit2] Song D., Jung Y. (2019). Angew. Chem., Int. Ed..

[cit3] Simon M. I., Strathmann M. P., Gautam N. (1991). Science.

[cit4] Lemmon M. A., Schlessinger J. (2000). Cell.

[cit5] Chen L., Marsiglia W. M., Chen H., Katigbak J., Erdjument-bromage H., Kemble D. J., Fu L., Ma J., Sun G., Zhang Y., Liang G., Neubert T. A., Li X., Traaseth N. J., Mohammadi M. (2020). Nat. Chem. Biol..

[cit6] Barton P., Hunter C. A., Potter T. J., Webb S. J., Williams N. H. (2002). Angew. Chem., Int. Ed..

[cit7] Schrader T., Maue M., Ellermann M. (2006). J. Recept. Signal Transduction Res..

[cit8] Ueki R., Atsuta S., Ueki A., Sando S. (2017). J. Am. Chem. Soc..

[cit9] Poli M. D., Zawodny W., Quinonero O., Lorch M., Webb S. J., Clayden J. (2016). Science.

[cit10] Kikuchi J., Ariga K., Ikeda K. (1999). Chem. Commun..

[cit11] Dijkstra H. P., Hutchinson J. J., Hunter C. A., Qin H., Tomas S., Webb S. J., Williams N. H. (2007). Chem.–Eur. J..

[cit12] Lister F. G. A., Bailly B. A. F. L., Webb S. J., Clayden J. (2017). Nat. Chem..

[cit13] Zadmard R., Schrader T. (2006). Angew. Chem., Int. Ed..

[cit14] Bernitzki K., Schrader T. (2009). Angew. Chem., Int. Ed..

[cit15] Bernitzki K., Maue M., Schrader T. (2012). Chem.–Eur. J..

[cit16] Langton M. J., Keymeulen F., Ciaccia M., Williams N. H., Hunter C. A. (2017). Nat. Chem..

[cit17] Langton M. J., Scriven L. M., Williams N. H., Hunter C. A. (2017). J. Am. Chem. Soc..

[cit18] Langton M. J., Williams N. H., Hunter C. A. (2017). J. Am. Chem. Soc..

[cit19] Ding Y., Williams N. H., Hunter C. A. (2019). J. Am. Chem. Soc..

[cit20] Birkholz O., Burns J. R., Richter C. P., Psathaki O. E., Howorka S., Piehler J. (2018). Nat. Commun..

[cit21] Langecker M., Arnaut V., Martin T. G., List J., Renner S., Mayer M., Dietz H., Simmel F. C. (2012). Science.

[cit22] Seifert A., Göpfrich K., Burns J. R., Fertig N., Keyser U. F., Howorka S. (2015). ACS Nano.

[cit23] Burns J. R., Stulz E., Howorka S. (2013). Nano Lett..

[cit24] Burns J. R., Howorka S. (2018). ACS Nano.

[cit25] Burns J. R., Seifert A., Fertig N., Howorka S. (2016). Nat. Nanotechnol..

[cit26] Burns J. R., Al-Juffali N., Janes S. M., Howorka S. (2014). Angew. Chem., Int. Ed..

[cit27] Burns J. R., Göpfrich K., Wood J. W., Thacker V. V., Stulz E., Keyser U. F., Howorka S. (2013). Angew. Chem., Int. Ed..

[cit28] Li C., Chen H., Zhou L., Shi H., He X., Yang X., Wang K., Liu J. (2019). Chem. Commun..

[cit29] Li C., Chen H., Chen Q., Shi H., Yang X., Wang K., Liu J. (2020). Anal. Chem..

[cit30] Vanuytsel S., Carniello J., Wallace M. I. (2019). ChemBioChem.

[cit31] Bekus R., Schrader T. (2020). ChemistryOpen.

[cit32] Alizadeh N., Salimi A., Hallaj R. (2020). Adv. Biochem. Eng./Biotechnol..

[cit33] Cox J. C., Ellington A. D. (2001). Bioorg. Med. Chem..

[cit34] Huizenga D. E., Szostak J. W. (1995). Biochemistry.

