# Supplementary material for: Controlled dimerization of artificial membrane receptors for transmembrane signal transduction
Source: Chem Sci. 2021 May 5;12(23):8224–30. doi: 10.1039/d1sc00718a (PMC8208304; doi:10.1039/d1sc00718a)
Supplement: SC-012-D1SC00718A-s001 [file SC-012-D1SC00718A-s001.pdf]

Electronic Supplementary Material (ESI) for Chemical Science.
This journal is © The Royal Society of Chemistry 2021

Supplementary Information

Controlled Dimerization of Artificial Membrane Receptors for Transmembrane Signal Transduction

Hui Chen, ‡ Li Zhou, ‡ Chunying Li, Xiaoxiao He, Jin Huang, Xiaohai Yang, Hui Shi,* Kemin Wang, Jianbo Liu*

State Key Laboratory of Chemo/Biosensing and Chemometrics, College of Biology, College of Chemistry and Chemical Engineering, Key Laboratory for Bio-Nanotechnology and Molecular Engineering of Hunan Province, Hunan University, Changsha 410082, P. R. China.

Table of content

Table S1. DNA sequences
Figure S1. Schematic diagram of the design of membrane receptors
Figure S2. Oligonucleotide sequences used in this work
Figure S3. Control experiment of the PAGE, CD spectra and fluorescence enhancement
Figure S4. The characterization of large unilamellar vesicle.
Figure S5. Investigation of the vesicle fusion and membrane mobility.
Figure S6. FRET efficiency comparison.
Figure S7. ATP mediated transmembrane signal transduction for ABTS oxidation.
Figure S8. Quantification of topological structure.
Figure S9. Lysozyme mediated dimerization in homogenous solution.

Table S1. DNA Sequences

Names	DNA Sequences (5'-3')
S1	TGGGTGGGTTTT/iSpC12/TTTACCTGGGGGAGTAT
S2	TGCGGAGGAAGGTTTT/iSpC12/TTTAGGGCGGGT
Sr	N_{13}TTT/iSpC12/TTTAGGGCGGGT
S3	TGCAGAGTTACTTAGTTTiSpC12/TTTAGGGCGGGT
S4	TGGGTGGGTTTT/iSpC12/TTTATCAGGGCTAAAGAG
FAM-S1	FAM-TGGGTGGGTTT/iSpC12/TTTACCTGGGGGAGTAT
FAM-Sc	FAM-TGGGTGGGTTTTCTTCAACATCTAGCTGGTGG
TAMRA-S2	TGCGGAGGAAGGTTTT/iSpC12/TTT-AGGGCGGGTTAMRA

Note: Orange sequences are the split G-quadruplex sequences, and blue sequences are the split ATP aptamer sequences. Pink sequences are the spilt lysozyme sequences. Green sequences are the random sequences and iSpC12 is C_{12} spacer.

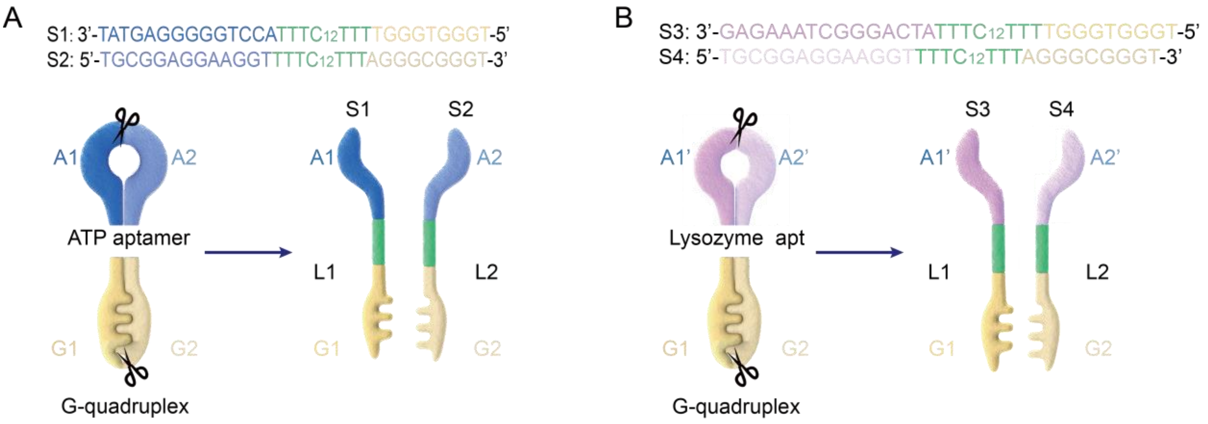

Figure S1. Schematic diagram of the design of membrane receptors. (A) The design of ATP responsive membrane receptors. Both S1 and S2 monomers were composed from three DNA modules. S1 monomer was composed from three DNA modules of A1, L1, and G1; and S2 monomer was composed from three DNA modules of A2, L2, and G2. While A1 and A2 are the split sequences of ATP aptamer, G1 and G2 are the split sequences of G-quadruplex. (B) The design of lysozyme responsive membrane receptors. Both S3 and S4 monomers were composed from three DNA modules. S3 monomer was composed from three DNA modules of A1', L1, and G1; and S4 monomer was composed from three DNA modules of A2', L2, and G2. While A1' and A2' are the split sequences of ATP aptamer, G1 and G2 are the split sequences of G-quadruplex. L1 and L2 are the C12-space-containing connecting sequences.

A

S1

Calculated MW	Measured MW	Relative error
9321.2	9322.2	0.01%

S2

Calculated MW	Measured MW	Relative error
9051.03	9051.8	0.009%

S3

Calculated MW	Measured MW	Relative error
9627.43	9627	0.004%

S4

Calculated MW	Measured MW	Relative error
9578.39	9578.5	0.001%

B

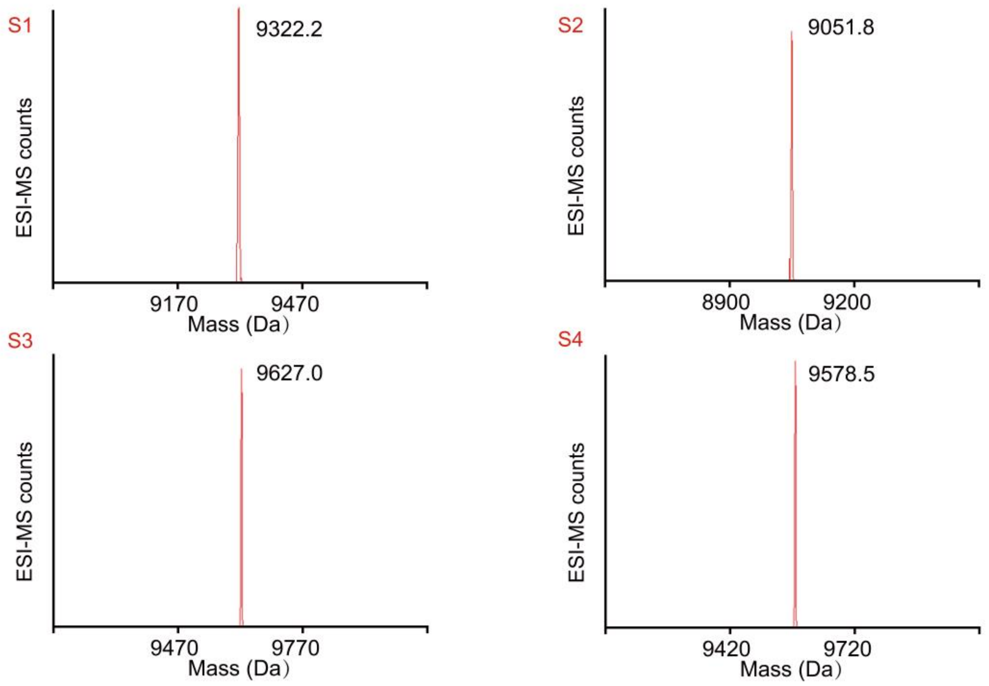

Figure S2. Oligonucleotide sequences used in this work: S1, S2, S3 and S4. (A) List of molecule weight of different DNA sequences. (B) ESI-MS for different DNA sequences. The molecular weights determined from ESI-MS were all consistent with the calculated values. Experimental conditions: DNA is dissolved in isopropanol solution, single electron, 10-2000 amu, Negative ion multi-charge mode.

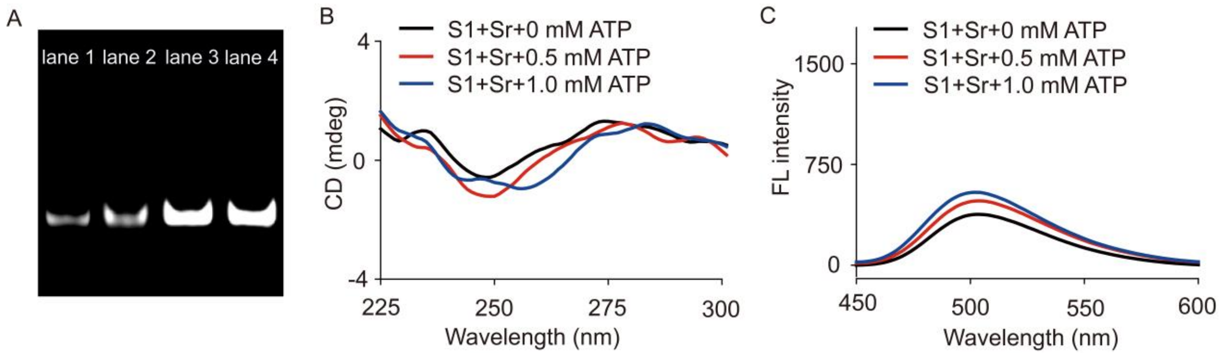

Figure S3. **Control experiment of the PAGE, CD spectra and fluorescence enhancement.** S2 was replaced by a random sequence of Sr. (A) PAGE gel electrophoresis, lane 1: S1; lane 2: Sr; lane 3: S1+Sr; lane 4: S1+Sr+ATP; (B) CD spectra of S1, Sr mixture in the presence of ATP; (C) Fluorescence enhancement of Thioflavin T (ThT) following binding to G-quadruplex.

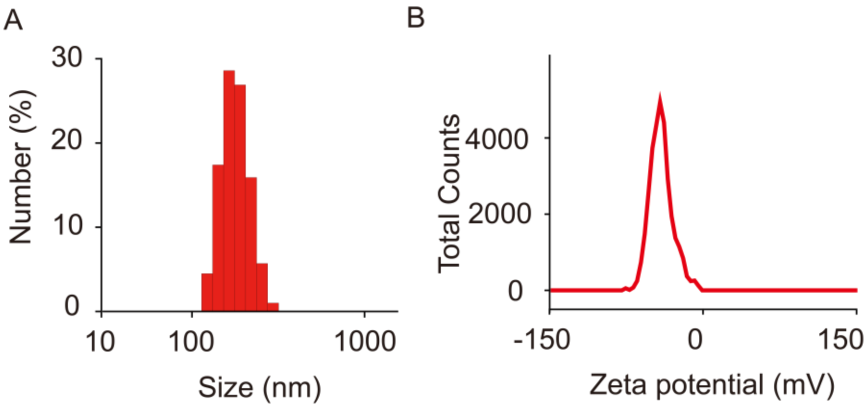

Figure S4. **The characterization of large unilamellar vesicle.** (A, B) Hydrodynamic size (A) and zeta potential (B) of LUV. pH=7.0. The diameters of the vesicles were determined by dynamic light scattering.

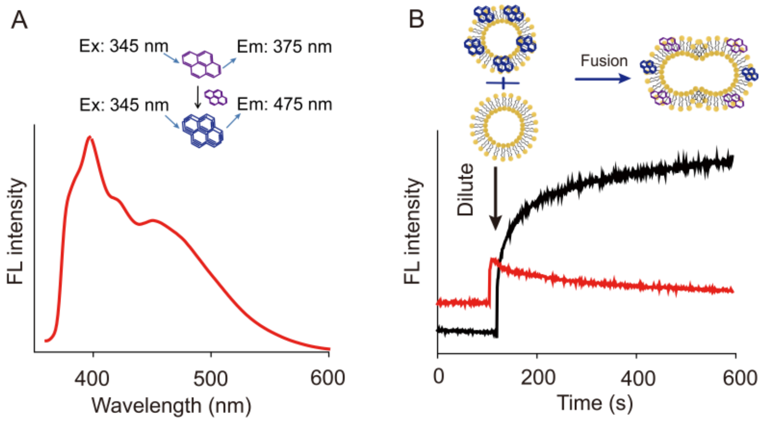

Figure S5. Investigation of the vesicle fusion and membrane mobility. (A) Fluorescence spectra of pyrene monomer at 375 nm and pyrene excimer at 475 nm. Ex: 345 nm; (B) Fluorescent kinetics curves of pyrene monomer (Black), and pyrene dimer (Red), when 50 µL pyrene-stained LUVs (0.5 mM pyrene) were added into equal volume of pyrene-free LUVs. The vesicle fusion and membrane mobility were characterized through pyrene fluorescence assay. 5 µL 5 mM pyrene solution was added into 50 µL LUV stock solution, resulting in the formation of pyrene-stained LUVs. The pyrene-stained LUVs were added into equal volume of pyrene-free LUVs, and incubated for 10 min. The fluorescence intensity of the pyrene monomer at 375 nm and the pyrene excimer at 475 nm were monitored after mixing with excitation at 345 nm.

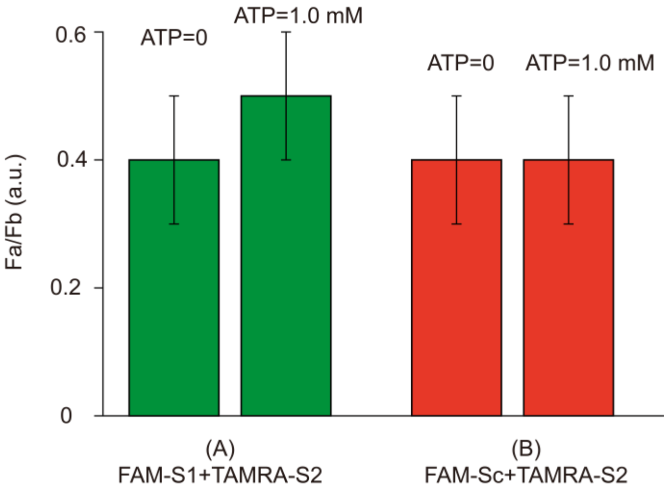

Figure S6. FRET efficiency comparison. (A) FRET experiments for FAM-S1 and TAMRA-S2 in the absence or presence of ATP (1.0 mM); (B) Control FRET experiments for FAM-Sc and TAMRA-S2 in the absence or presence of ATP (1.0 mM). As a control experiment, FAM-S1 was replaced by random sequences-containing FAM-Sc.

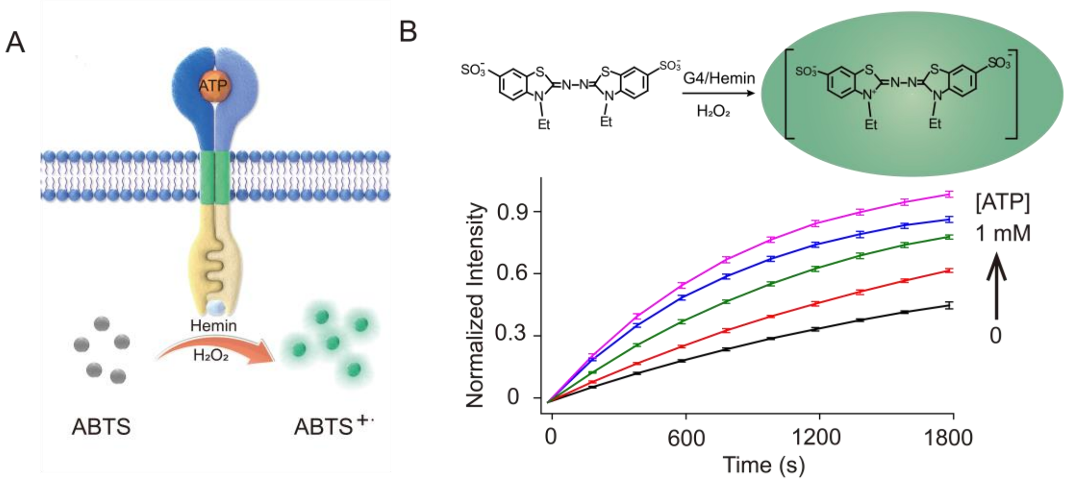

Figure S7. ATP mediated transmembrane signal transduction for ABTS oxidation. (A) Schematic illustration of ATP input triggered dimer formation and G4-hemin mediated ABTS oxidation; (B) UV-vis adsorption kinetic curve of ABTS oxidized by G4-hemin after addition of ATP (0-1.0 mM). Inset: a scheme of the G4-hemin mediated ABTS oxidation by H_2O_2. S1: S2=1:1 in molar. H_2O_2: 50 µM, hemin: 4.0 µM, ABTS: 2 mM, Abs: 420 nm.

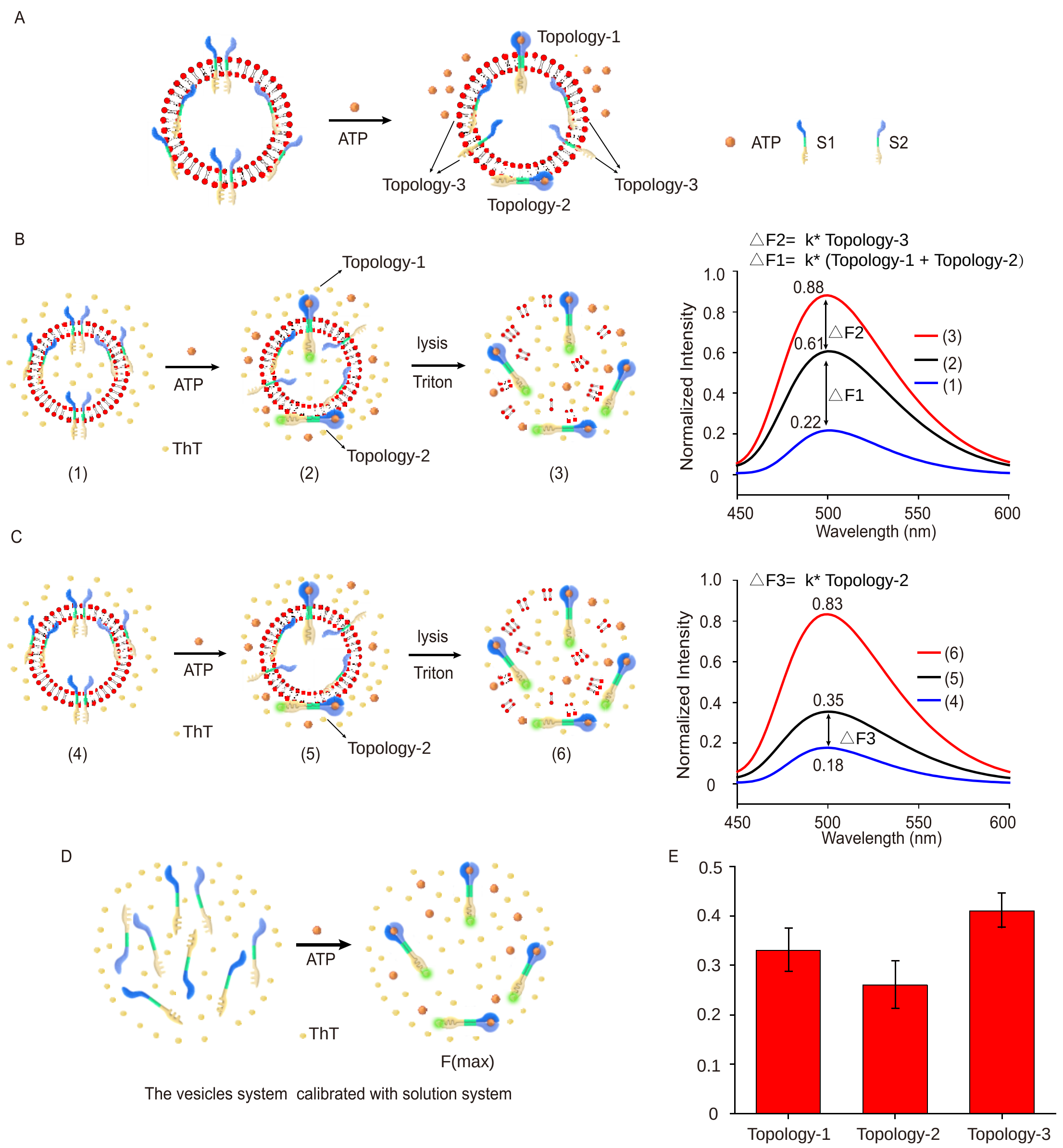

Figure S8. Quantification of possible topological structures. (A) Schematic diagram of possible topological structures in vesicles without and with addition of ATP; Topology-1: dimer in the outer leaflet; Topology-2: monomer in the outer leaflet; Topology-3: dimer and monomer in the inner leaflet. Two fluorescence vesicle experiments (B & C) have been conducted, based on the fluorescence enhancement of thioflavin T (ThT) upon binding with G-quadruplex. (B) Fluorescence vesicle experiment: ThT is filled in the interior and exterior of the vesicles. Addition of ATP triggers the dimerization with Topology-1 & -2 structures, and their corresponding fluorescence enhancement (ΔF1). Upon addition of 1% Triton leads to the dimerization with Topology-3 structures, and their corresponding fluorescence enhancement (ΔF2). (C) Fluorescence vesicle experiment: ThT is filled in the exterior of the vesicles. Addition of ATP triggers the dimerization with Topology-1 & -2 structures, but only fluorescence enhancement (ΔF3) for Topology-2 structures. Upon addition of 1% Triton leads to the dimerization with Topology-3 structures, and fluorescence enhancement for Topology-1 & -3 structures. (D) The fluorescent intensity in B & C was calibrated with the maximum fluorescence value of the dyes in non-vesicle system. The concentrations of S1, S2, ATP, ThT are the same in B, C and D; (E) The fractions of the different topologies in vesicles. The transmembrane units in the outer leaflet in the correct orientation (Topology-1) only accounted for 33%.

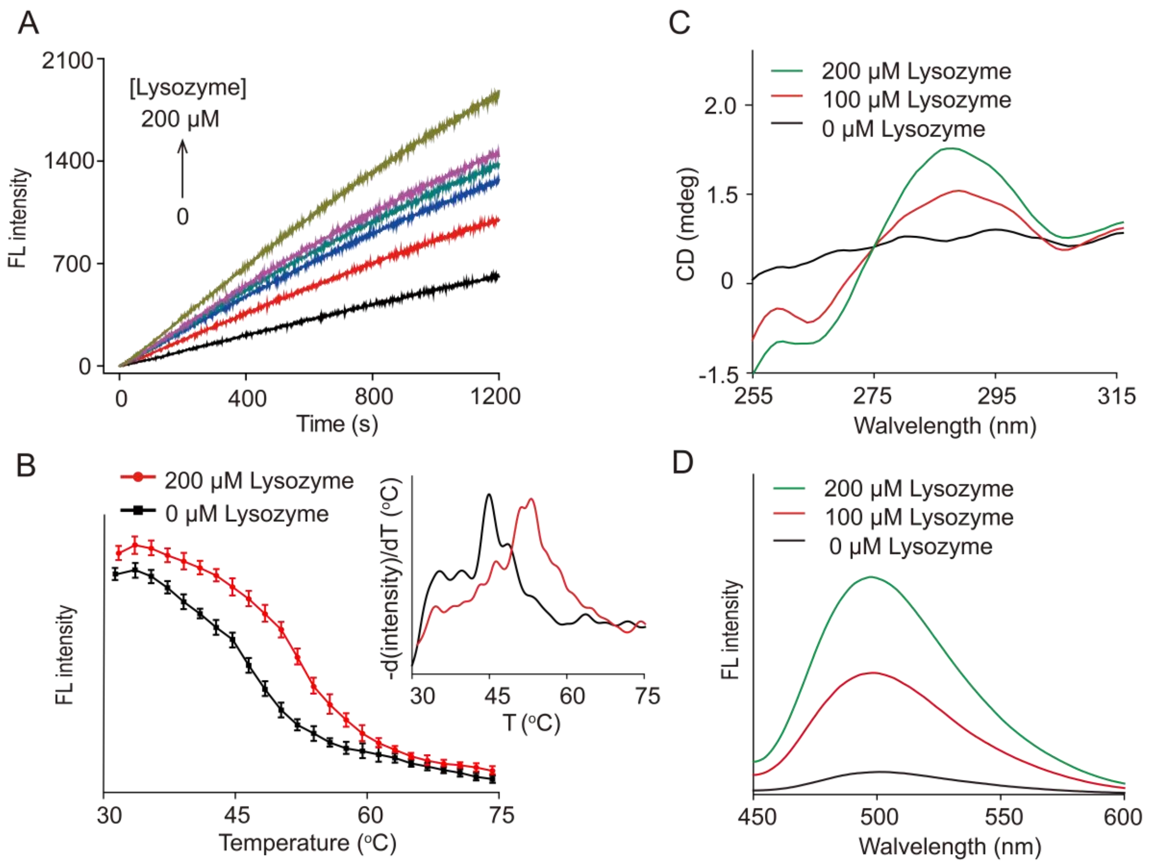

Figure S9. Lysozyme mediated dimerization in homogenous solution. (A) Fluorescent kinetic curve of Amplex red oxidized by G4-hemin after addition of lysozyme (0-200 μM) in homogenous solution. S3: S4=1:1 in molar. H_2O_2: 50 μM, hemin: 4.0 μM, Amplex red: 20 μM. (B) Melting curve of the split sequences mixture (S3 and S4) in the absence or presence of lysozyme (200 μM). The fluorescence intensity of the split sequences mixture with ThT decreased with increasing temperature; (Inset) Negative first derivative of the melting curves. (C) CD spectra of S3 and S4 mixture in the different concentrations of lysozyme (0-100 μM). (D) Fluorescence enhancement of ThT following binding to G-quadruplex, formed by lysozyme (0-100 μM) mediated dimerization of split sequences.

Our results show that lysozyme can facilitate a signal output. CD spectra show that the dimer dimerization accelerate a formation of G-quadruplex structure. The melting curves of the mixed split sequences indicate that addition of lysozyme facilitated an increase in the temperature of melting (Tm) value of the DNA complex from 45.0 ℃ to 51.0 ℃ (**Figure S9B**). Gradual addition of lysozyme into the split sequences mixture induced an appearance of a negative peak around 260 nm and a positive peak around 290 nm, corresponding to a characteristic peak of G-quadruplex antiparallel structure (**Figure S9C**). The formation of G-quadruplex was further confirmed by ThT fluorescence. It was showed that fluorescent emission of ThT at 504 nm increased 9.4-fold after addition of lysozyme (100 μM), which was contributed to the formation of G-quadruplex (**Figure S9D**). These results demonstrated that lysozyme input triggered dimerization is accompanied by G-quadruplex formation, which could serve as peroxidase-like enzyme for signal production.
